# Refinement-to-Coarsening Transition During Multiple Quenching of SCM440 Steel: Competing Roles of Austenite Reformation and Carbide Dissolution

**DOI:** 10.3390/ma19214572

**Published:** 2026-10-29

**Authors:** Byoungho Choi, Jihun Shin, Kyusun Kang, Kyoung Il Moon, Gi-Hoon Kwon

**Affiliations:** 1 Customized Manufacturing R&D Department (Siheung Technology Support Center), Korea Institute of Industrial Technology, Siheung 15014, Republic of Korea; 2 R&D Department, Jangin Metal Co., Ltd., Siheung 15091, Republic of Korea

**Keywords:** SCM440 steel, multiple quenching, prior-austenite grain size, austenite reformation, Zener pinning

## Abstract

This study investigated the grain refinement-to-coarsening transition in SCM440 steel during multiple quenching, focusing on repeated austenite reformation, carbide dissolution, and grain-boundary migration. Specimens were austenitized at 850 °C for 20 min and water-quenched for one to four cycles (AQ-1–AQ-4), while AH-80 was continuously held at 850 °C for 80 min and quenched once. The prior-austenite grain size decreased from 12.2 ± 4.6 μm in AQ-1 to 8.5 ± 2.5 μm in AQ-3, but increased to 15.2 ± 4.1 μm in AQ-4 and 22.6 ± 5.4 μm in AH-80. EBSD showed that AQ-3 also exhibited the minimum effective grain size of 0.78 μm and the highest average KAM of 1.62°. TEM revealed a progressive reduction in the carbide population observed after quenching, with the projected area fraction decreasing from 7.39% in AQ-1 to 0.23% in AH-80, consistent with progressive carbide dissolution during austenitization. Based on simplified assumptions regarding carbide geometry, volume fraction, and grain-boundary energy, the estimated Zener pinning pressure decreased from 0.429 to 0.045 MPa, while the estimated PZ/PD ratio decreased from 2.616 to approximately 0.51. Despite progressively weakened carbide pinning, grain refinement continued up to AQ-3, indicating that enhanced pinning alone cannot explain the refinement and suggesting an additional contribution from repeated austenite reformation. AQ-3 exhibited the highest yield strength and ultimate tensile strength and Charpy impact energy, whereas subsequent grain coarsening reduced the mechanical performance. These findings are consistent with a possible competition among repeated austenite reformation, residual-carbide pinning, and grain-boundary migration, although further time-resolved investigations are required to validate their relative contributions.

## 1. Introduction

Medium-carbon Cr–Mo low-alloy steels are widely used for highly loaded engineering components because of their high hardenability and favorable combination of strength, toughness, wear resistance, and fatigue performance. Among these steels, SCM440 steel, specified in JIS G 4053, is widely employed for shafts, gears, fasteners, and other structural components requiring high mechanical reliability. Its mechanical performance is strongly dependent on austenitizing and quenching conditions that determine the prior-austenite grain (PAG) structure and subsequent martensitic transformation. The prior-austenite grain size (PAGS) affects the hierarchical martensitic structure consisting of packets, blocks, and laths, with PAGS refinement generally accompanied by refinement of the martensitic substructure and changes in high-angle boundary density [1,2]. These crystallographic subdivisions influence dislocation motion and crack propagation and therefore play an important role in the strength and toughness of martensitic steels [1,2,3]. PAG refinement is thus an effective strategy for controlling the mechanical performance of quenched steels. However, finer austenite grains increase the grain-boundary area per unit volume and the capillary tendency for subsequent grain growth. Maintaining a refined PAG structure therefore requires consideration of both the formation of fine austenite and the restriction of subsequent grain-boundary migration.

Repeated austenitizing and quenching, also referred to as cyclic or multiple quenching, provides a practical route for refining the austenitic and martensitic structures without changing the nominal alloy composition. In AISI 4140 steel, Khani Sanij et al. demonstrated that double quenching refined prior-austenite grains and martensitic packets and improved impact toughness compared with conventional single quenching [4]. Smoljan similarly reported increased yield strength and Charpy toughness following cyclic heat treatment of AISI 4140 and related these improvements to structural refinement [5]. Recent work on medium-carbon Cr–Ni–Mo–V steel showed that multiple quenching modifies PAGS, martensitic lath dimensions, high-angle grain-boundary density, and variant selection, thereby affecting mechanical behavior [6]. Similar refinement through repeated or modified austenitizing treatments has been reported in other martensitic and ultrahigh-strength steels [7,8,9,10]. Multi-pass thermal cycling of normalized AISI 4140 was also shown to refine the transformed structure while homogenizing the initially heterogeneous carbon distribution [11]. These studies establish repeated phase transformation as an effective route for microstructural refinement; however, they do not demonstrate that increasing the number of cycles necessarily produces continuous grain refinement. During re-austenitization, prior-austenite grain boundaries, martensitic interfaces, and defects may promote austenite nucleation and grain refinement. Residual carbides may influence local carbon availability during transformation while restricting subsequent grain-boundary migration through Zener pinning.

Multiple quenching involves competing processes of austenite reformation and grain growth during each thermal cycle. A distinction can therefore be made between mechanisms that generate a refined austenitic structure during re-austenitization and those that preserve it by restricting grain-boundary migration. Residual carbide particles can contribute to the latter through Zener pinning, while their population progressively changes during repeated austenitizing. Studies of 42CrMo4 steel have shown that carbide dissolution during austenitization involves redistribution of carbon and substitutional alloying elements and can modify austenite chemistry [12]. Multiple-quenching experiments on carbide-rich tool steels have similarly demonstrated concurrent changes in carbide characteristics and austenite grain size; notably, partial grain coarsening beyond the third cycle was reported in D2 steel [13]. Multiple quenching of SAE52100 has also been reported to decrease PAGS while increasing retained-austenite content [14]. Thus, multiple quenching cannot be interpreted solely as a cumulative grain-refinement process because the particle population restricting grain-boundary migration evolves simultaneously with repeated phase transformation.

This competition becomes increasingly important as the austenite structure is refined. As carbide pinning weakens, grain coarsening is expected to occur primarily through normal grain growth driven by grain-boundary migration. However, abnormal grain growth may also occur if local variations in carbide distribution lead to heterogeneous boundary mobility. From a capillarity perspective, decreasing grain size increases the driving pressure associated with grain-boundary curvature, whereas progressive carbide dissolution can reduce the opposing Zener pinning pressure. Consequently, the refined structure produced by repeated austenite reformation may eventually become susceptible to subsequent grain-boundary migration. Austenite grain-growth studies of AISI 4140 have shown that increasing austenitizing temperature and holding time promotes grain growth [15], while rapid austenitization studies have demonstrated the interplay among holding time, carbide dissolution, PAGS, and mechanical properties [16]. Cyclic heat-treatment studies have also established that repeated austenite–martensite transformations can substantially refine initially coarse austenite grains [17]. Nevertheless, the origin of the refinement-to-coarsening transition during successive quenching cycles remains insufficiently understood. In particular, it remains unclear why PAG refinement can persist while the residual carbide population progressively decreases and when re-austenitization-induced refinement becomes insufficient to counteract capillary-driven grain-boundary migration. Moreover, the relative contributions of particle-mediated pinning and capillary driving pressure have rarely been quantitatively considered in interpreting multiple-quenching-induced PAG evolution. Separating these contributions also requires distinguishing repeated transformation effects from the simple accumulation of austenitizing time.

We hypothesize that repeated re-austenitization initially promotes prior-austenite grain refinement, whereas progressive carbide dissolution weakens Zener pinning and eventually facilitates grain coarsening. To examine this hypothesis, SCM440 steel was subjected to one to four multiple quenching cycles at 850 °C for 20 min per cycle, with an additional specimen continuously held for 80 min (AH-80) to distinguish repeated transformation effects from cumulative thermal exposure. Microstructural evolution was characterized using optical microscopy (OM), scanning electron microscopy (SEM), electron backscatter diffraction (EBSD), transmission electron microscopy (TEM), and X-ray diffraction (XRD), and correlated with mechanical properties. The competition between carbide pinning and grain-boundary migration was evaluated by comparing estimated Zener pinning and capillary driving pressures.

The novelty of this study lies in two contributions: (i) distinguishing the effects of repeated austenite reformation from cumulative thermal exposure by comparing AQ-4 and AH-80 under equivalent austenitizing times, and (ii) quantitatively comparing the estimated Zener pinning pressure of residual carbides with the capillary driving pressure for grain growth. Together, these approaches provide insight into the refinement-to-coarsening transition during multiple quenching of SCM440 steel.

## 2. Experimental Procedure

### 2.1. Materials and Heat Treatment

Commercial SCM440 medium-carbon Cr–Mo low-alloy steel (manufactured by SeAH Besteel, Gunsan, Republic of Korea) in the hot-rolled and annealed condition was used. Cylindrical specimens with a diameter of 60 mm and a height of 10 mm were prepared for heat treatment. Its chemical composition was determined by spark optical emission spectroscopy (Spark-OES; SPECTROMAXx LMX09, SPECTRO Analytical Instruments, Kleve, Germany) and is listed in Table 1.

Austenitization was performed in a cylindrical horizontal quartz-tube furnace with a hot zone of 100 mm in diameter and 260 mm in length. The furnace was evacuated to high vacuum using a turbomolecular pumping system and subsequently backfilled with high-purity N_2_ to 760 Torr to minimize oxidation and decarburization. The specimens were heated to 850 °C at 10 °C min^−1^, held for 20 min, and water-quenched to room temperature. The average cooling rate was approximately 170 °C s^−1^ over 850–500 °C.

**Figure 1:**
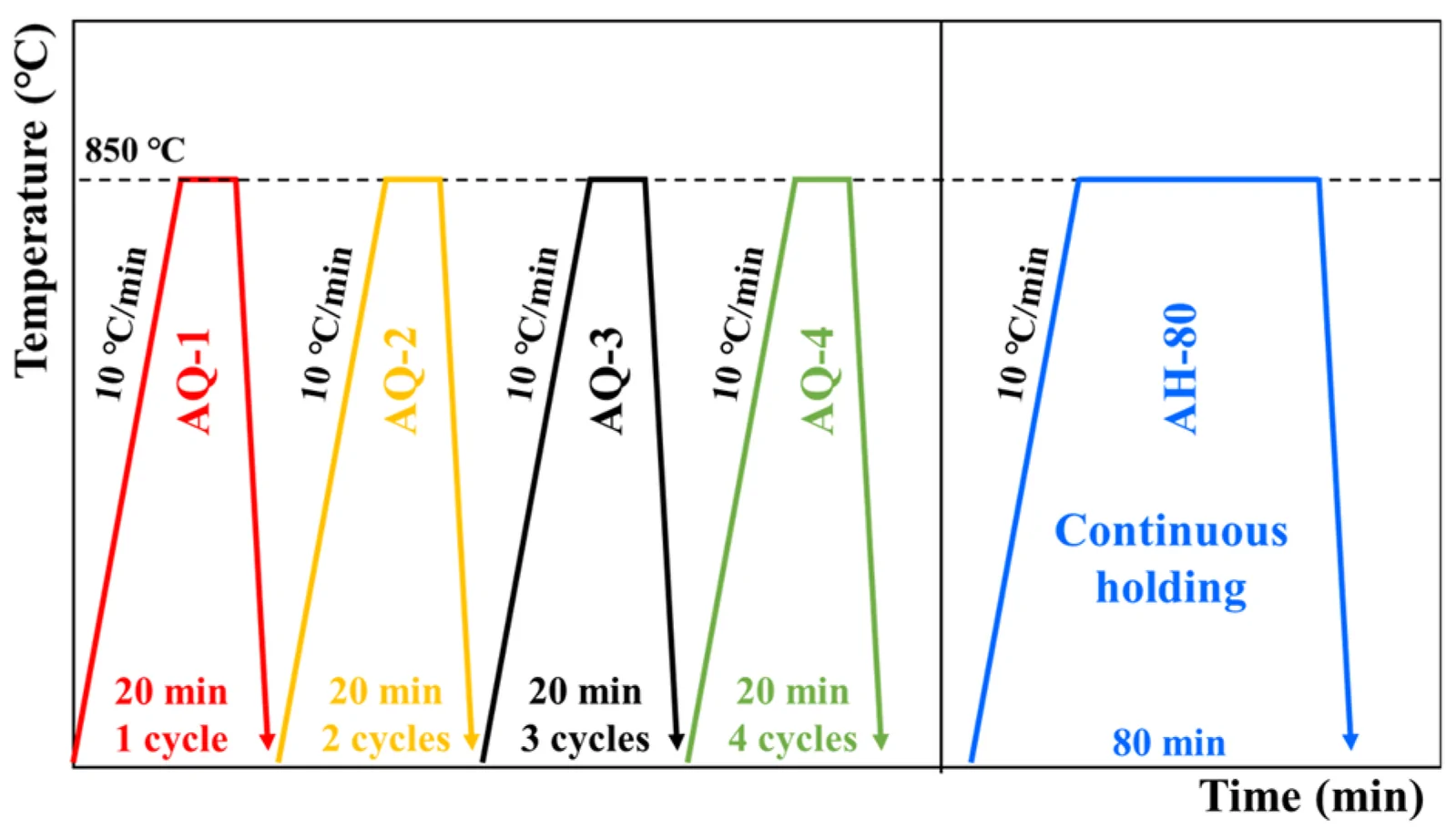
Schematic illustration of the heat-treatment schedules for SCM440 steel subjected to multiple cycles (AQ-1–AQ-4) and continuous austenitizing holding (AH-80).

This cycle was repeated one to four times, with the specimens designated AQ-1, AQ-2, AQ-3, and AQ-4, respectively. Each subsequent cycle consisted of reheating the quenched specimen to 850 °C, holding for 20 min, and water quenching under identical conditions. After each quenching cycle, the specimens were allowed to return to room temperature and held for approximately 20 min before reheating. The same specimens were subjected to successive austenitizing and quenching cycles according to the designated cycle number. For comparison, AH-80 was continuously held at 850 °C for 80 min and water-quenched once. Thus, AQ-4 and AH-80 had the same cumulative austenitizing time (80 min) but differed in the presence of intermediate quenching and re-austenitization. Five independent specimens were prepared for each heat-treatment condition (AQ-1–AQ-4 and AH-80) to ensure the reproducibility of the experimental results. The heat-treatment schedules are illustrated in Figure 1.

### 2.2. Microstructural Characterization

Specimens for microstructural characterization were ground with SiC papers from #300 to #2000 grit and polished using diamond suspension. The polished specimens were etched with 4 vol.% nital for 10 s to reveal the martensitic microstructure, followed by 4 vol.% picral for 20 s to enhance carbide contrast. Microstructures were examined by optical microscopy (OM; HRM-300, MTDI Inc., Daejeon, Republic of Korea) and scanning electron microscopy (SEM; NNS-450, FEI, Hong Kong, China). Prior-austenite grain boundaries were revealed by conventional metallographic etching, and the prior-austenite grain size (PAGS) was determined using the linear-intercept method [18] from 10 optical micrographs per condition, with a total of 300 grain-boundary intercepts measured. The results are reported as the mean ± standard deviation.

Electron backscatter diffraction (EBSD; Velocity Super, EDAX, Paoli, PA, USA) was used to characterize the martensitic substructure rather than the prior-austenite grain size. Orientation maps were acquired at a 20 nm step size over an area of approximately 15 × 11 μm^2^ with five observation fields analyzed per condition. Boundaries with misorientations of 2–15° and >15° were classified as low-angle (LAGBs) and high-angle grain boundaries (HAGBs), respectively. Inverse pole figure (IPF), grain-boundary, and kernel average misorientation (KAM) maps were obtained. Effective grain size was determined by the area-weighted mean-intercept method using >15° boundaries, while KAM was calculated using first nearest-neighbor pixels with a 5° misorientation cutoff. Isolated misindexed pixels were corrected using wild-spike removal, and the indexing rate was defined as the percentage of successfully indexed pixels. For EBSD analysis, the specimens were additionally polished using a 0.04 μm colloidal silica suspension for 10 min to minimize surface deformation and improve the quality of diffraction patterns.

The martensitic substructure and residual carbides were examined by transmission electron microscopy (TEM; Tecnai G2 F20, FEI Company, Hillsboro, OR, USA) at 200 kV using mechanically thinned and electropolished foils. Bright-field TEM was used to characterize martensitic laths and residual carbides, while high-resolution transmission electron microscopy (HRTEM) analysis was used for crystallographic characterization of selected carbide/matrix regions. Residual carbide size, area fraction, and number density were quantitatively determined from TEM micrographs by image analysis. The equivalent circular diameter of each carbide was calculated as:

$$d_{eq}=2\sqrt{\frac{A}{\pi }}$$

where *A* is the projected particle area. The mean carbide size was obtained from the measured particle population, while the carbide area fraction ($f_{A}$) was determined as the total projected carbide area divided by the analyzed TEM image area. The number density ($N_{A}$) was calculated as the number of identified carbide particles per unit analyzed area. The measured two-dimensional area fraction was used as an approximation of the carbide volume fraction ($f_{V}$ ≈ $f_{A}$) under a stereological assumption of representative particle sampling [19]. Quantitative carbide analysis was performed using five low-magnification TEM micrographs acquired from independent observation fields for each heat-treatment condition (*n* = 5). TEM thin-foil measurements may involve sectioning and particle-visibility biases despite representative sampling. Thus, the estimated carbide volume fractions and Zener pinning pressures should be considered semi-quantitative. For statistical analysis, the individual carbide measurements from the five observation fields were pooled to obtain the overall particle size distribution and mean equivalent-circle diameter. The projected area fraction and areal number density were calculated by dividing the total detected carbide area and particle count, respectively, by the total analyzed TEM image area.

To evaluate the relative resistance of residual carbides to austenite grain-boundary migration, the Zener pinning pressure ($P_{Z})$ was estimated according to the classical Zener relationship [20]:

$$P_{Z}=\frac{3f\gamma_{GB}}{2r}$$

where f is the estimated carbide volume fraction, *r* is the mean carbide radius, and $\gamma_{GB}$ is the austenite grain-boundary energy. A value of 0.5 J m^−2^ was adopted for $\gamma_{GB}$, consistent with values used for austenite grain-growth calculations in low-alloy steels [20]. This literature-based value was held constant across all conditions and excluded from the propagation of experimental measurement uncertainties. The capillary driving pressure for grain growth, $P_{D}$, was estimated as

$$P_{D}=\frac{4\gamma_{GB}}{D}$$

where *D* is the experimentally measured final PAGS. The relationship $P_{D}$ = 4$\gamma_{GB}$/*D* provides a simplified estimate of capillary pressure based on idealized grain geometry, neglecting variations in boundary curvature and energy. Since the final PAGS was used, $P_{D}$ represents an estimated post-treatment capillary pressure rather than an independent predictor of grain growth. Accordingly, the $P_{z}$/$P_{D}$ ratio provides only a qualitative comparison of pinning and capillary pressures, with additional uncertainty arising from the TEM-based estimation of carbide volume fraction.

Phase evolution was analyzed by X-ray diffraction (XRD; MiniFlex II, Rigaku, Tokyo, Japan) using Co Kα radiation (*λ* = 1.789 Å). Diffraction patterns were recorded over 40–120° (2θ) with a step size of 0.02° and a scan rate of 2° min^−1^. The retained-austenite fraction was determined from the integrated intensities of selected martensite and austenite reflections, with identical acquisition and fitting procedures applied to all conditions [21].

$$V_{\gamma }=\frac{I_{\gamma }/R_{\gamma }}{I_{\gamma }/R_{\gamma }+I_{\alpha^{\prime }}/R_{\alpha^{\prime }}}$$

The retained-austenite volume fraction (*V_γ_*) was calculated from the corrected integrated intensities of the martensite (*α*′) and austenite (*γ*) reflections using the following equation. Here, *I*/*R* represents the mean corrected integrated intensity of the selected reflections for each phase, where *I* is the background-corrected integrated peak intensity and *R* is the theoretical intensity correction factor accounting for the structure factor, multiplicity, and Lorentz–polarization effects.

### 2.3. Mechanical Characterization

Mechanical properties were evaluated by tensile, Rockwell hardness, and Charpy impact tests. Room-temperature tensile tests were conducted using a universal testing machine (5982, Instron Corp., Norwood, MA, USA) in accordance with ASTM E8/E8M. The specimens were machined parallel to the rolling direction with a gauge length of 25 mm, a gauge width of 6 mm, and a thickness of 5 mm. An extensometer with a gauge length of 25 mm was used to measure strain. The initial strain rate was approximately 1 × 10^−3^ s^−1^. Yield strength was determined by the 0.2% offset method, and at least three specimens were tested for each condition. Rockwell C hardness was measured five times per specimen (Ø60 mm) using an LC-200RB tester (Future-Tech Corp., Kawasaki, Japan) under a 150 kgf load in accordance with ASTM E18. The indentation spacing and edge distance were maintained at ≥3d and ≥2.5d, respectively, where d is the indentation diameter. The average hardness was reported.

Charpy V-notch impact tests (SI-1D3, Instron Corp., Norwood, MA, USA) were conducted using specimens with their longitudinal axes oriented perpendicular to the rolling direction in accordance with ASTM E23. Standard specimens (55 × 10 × 10 mm^3^) with a 2-mm-deep, 45° V-notch were used, and at least three specimens were tested for each condition. Following impact testing, representative fracture surfaces from all five heat-treatment conditions (AQ-1, AQ-2, AQ-3, AQ-4, and AH-80) were examined by SEM to correlate fracture morphology with impact behavior.

## 3. Results and Discussion

### 3.1. Microstructural Evolution During Multiple Quenching

**Figure 2:**
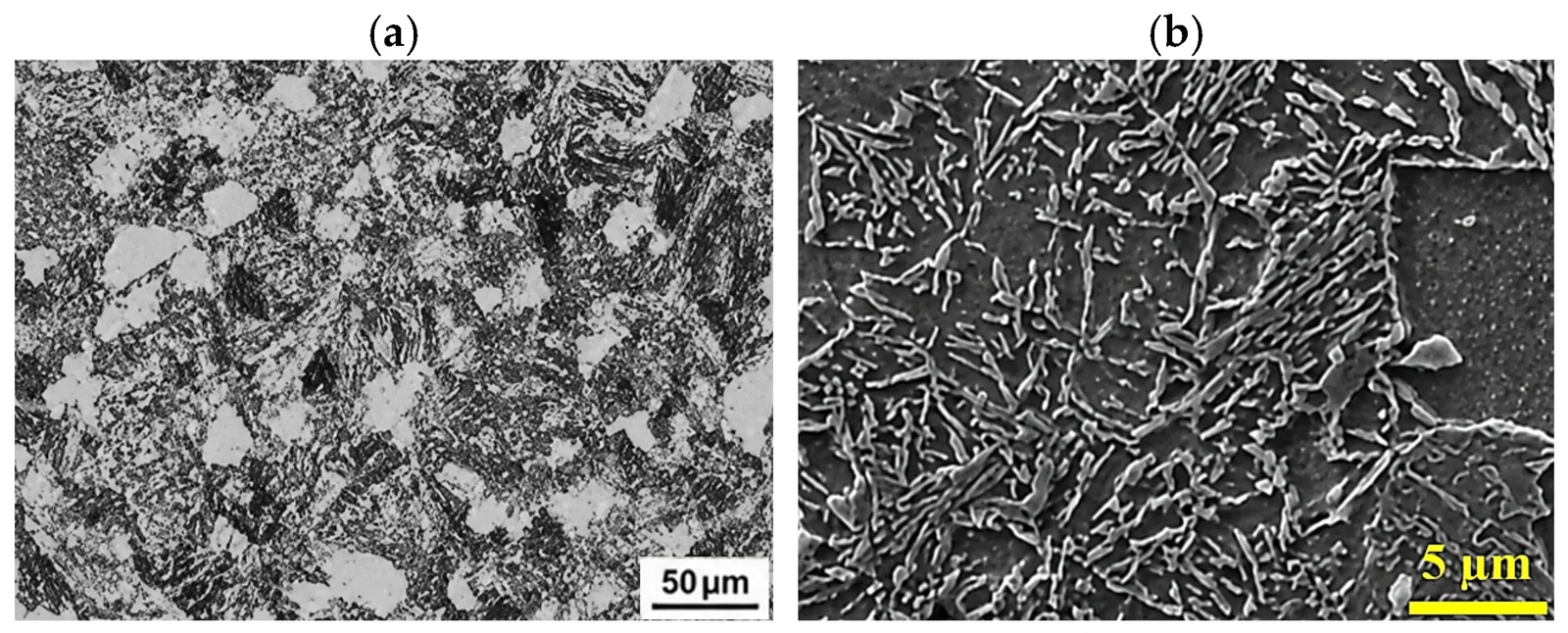
Initial microstructure of the hot-rolled and annealed SCM440 steel: (**a**) optical and (**b**) SEM micrographs.

Figure 2 shows the initial microstructure of the hot-rolled and annealed SCM440 steel. The optical micrograph in Figure 2a reveals a typical ferritic–pearlitic microstructure of annealed medium-carbon Cr–Mo steel. At higher magnification, the SEM image in Figure 2b shows numerous elongated and partially fragmented cementite/carbide constituents distributed within the ferritic matrix. Similar ferrite–pearlite structures and the evolution of lamellar cementite toward granular or spheroidized morphologies have been reported in annealed SCM440 and compositionally comparable 42CrMo4 steels [22].

**Figure 3:**
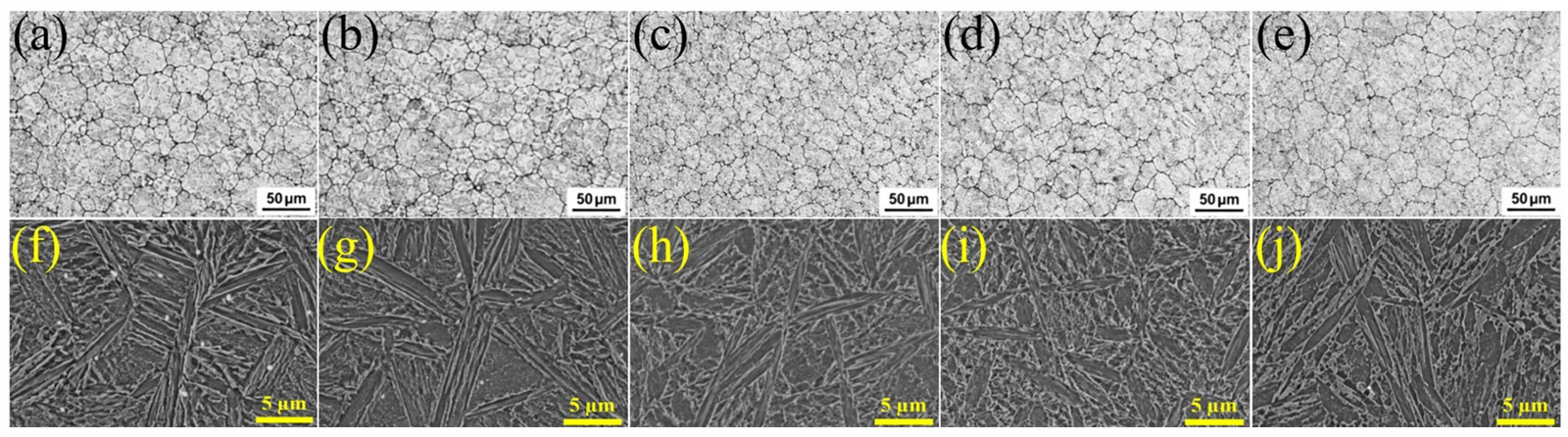
Evolution of prior-austenite grain structure, martensitic microstructure, and residual carbide morphology in SCM440 steel subjected to multiple quenching: (**a**–**e**) prior-austenite grain structures and (**f**–**j**) corresponding SEM micrographs of martensitic microstructures and residual carbides for AQ-1, AQ-2, AQ-3, AQ-4, and AH-80, respectively.

Figure 3 shows the evolution of prior-austenite grain structure, martensitic microstructure, and residual carbide morphology during multiple quenching. The PAGS decreased from 12.2 ± 4.6 μm in AQ-1 to 10.5 ± 3.0 μm in AQ-2 and reached a minimum of 8.5 ± 2.5 μm in AQ-3, before increasing to 15.2 ± 4.1 μm in AQ-4, indicating a refinement-to-coarsening transition. AH-80 exhibited the coarsest prior-austenite structure, with a PAGS of 22.6 ± 5.4 μm. Although AQ-4 and AH-80 experienced the same cumulative austenitizing time of 80 min at 850 °C, AQ-4 retained finer grains. This difference suggests that PAGS evolution is not governed solely by cumulative holding time and may be influenced by intermediate quenching and re-austenitization. The coarsening from AQ-3 to AQ-4 suggests that the refinement effect became insufficient to counteract grain growth during the fourth cycle.

The corresponding SEM micrographs in Figure 3 reveal predominantly lath-like martensitic structures with variations in morphology and residual carbide distribution. Discrete carbide particles were observed in AQ-1 and AQ-2 but became increasingly difficult to distinguish at the SEM scale from AQ-3 onward, consistent with progressive carbide dissolution during repeated austenitizing at 850 °C. However, the absence of visible carbides does not indicate complete dissolution, as finer particles may remain below the SEM resolution limit. These features were further examined using EBSD and TEM to characterize the martensitic substructure and nanoscale residual carbides, respectively.

**Figure 4:**
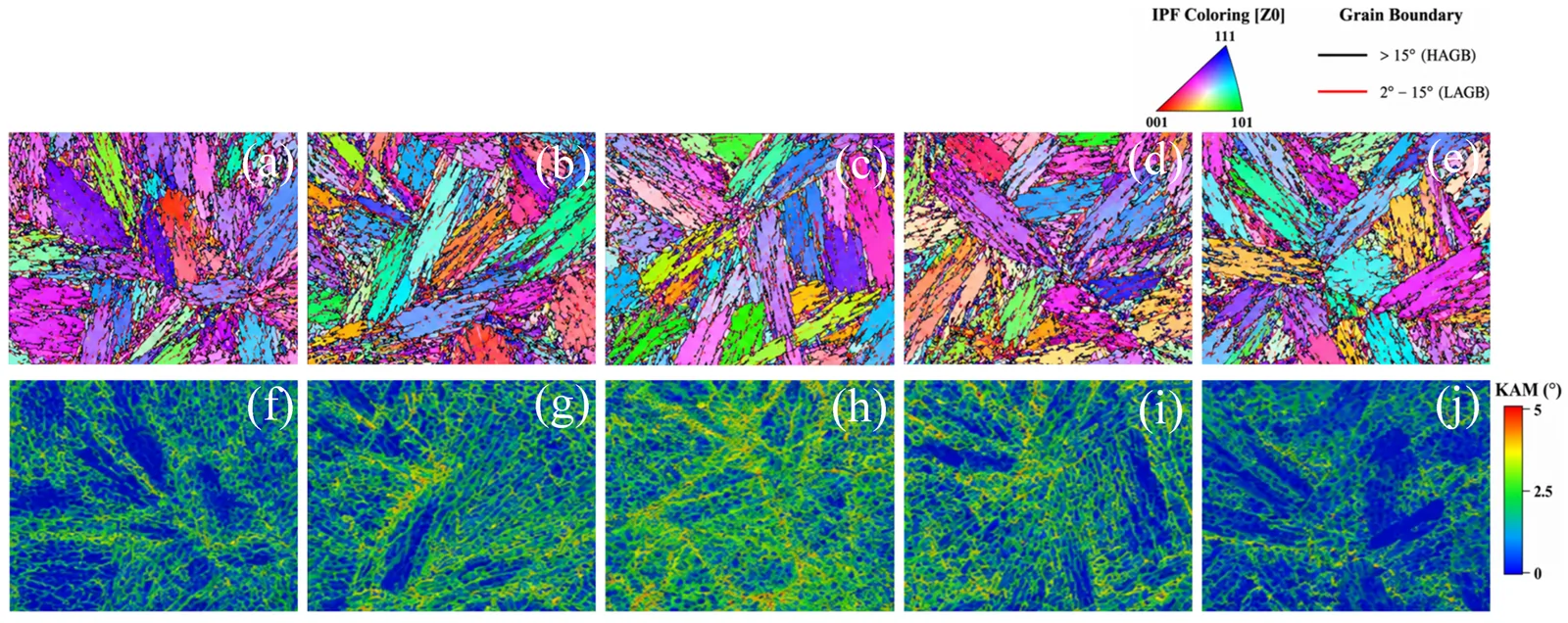
EBSD characterization of SCM440 steel subjected to multiple quenching: (**a**–**e**) IPF maps overlaid with grain-boundary maps and (**f**–**j**) KAM maps for AQ-1, AQ-2, AQ-3, AQ-4, and AH-80, respectively. HAGBs (>15°) and LAGBs (2–15°) are indicated by black and red lines, respectively.

Figure 4 presents representative EBSD IPF–grain boundary and KAM maps of the specimens subjected to multiple quenching, while the quantitative EBSD results are summarized in Table 2.

The martensitic structure became progressively refined from AQ-1 to AQ-3, as reflected by the decrease in effective grain size from 1.28 ± 0.14 to 0.78 ± 0.09 μm. Concurrently, the average KAM increased from 1.05 ± 0.12° to 1.62 ± 0.16°, while the HAGB fraction increased from 35.2 ± 3.1% to 45.8 ± 3.7%. These observations suggest that the minimum PAGS in AQ-3 was accompanied by a more highly subdivided martensitic structure and increased local crystallographic misorientation. However, KAM is influenced by EBSD step size, measurement noise, and data processing, and therefore should not be directly interpreted as a quantitative measure of dislocation density.

After the fourth cycle, the effective grain size slightly increased to 0.85 ± 0.10 μm, while the average KAM and HAGB fraction decreased to 1.44 ± 0.15° and 42.1 ± 3.5%, respectively. These changes were more pronounced in AH-80, which exhibited the largest effective grain size (1.55 ± 0.17 μm), the lowest average KAM (0.88 ± 0.10°), and the lowest HAGB fraction (32.4 ± 2.9%). The LAGB fraction exhibited an opposite trend to the HAGB fraction, decreasing to 54.2 ± 3.7% in AQ-3 before increasing in AQ-4 and AH-80. Overall, the EBSD results show a trend broadly consistent with the non-monotonic PAGS evolution in Figure 3, with the finest effective grain size and highest local misorientation observed in AQ-3.

**Figure 5:**
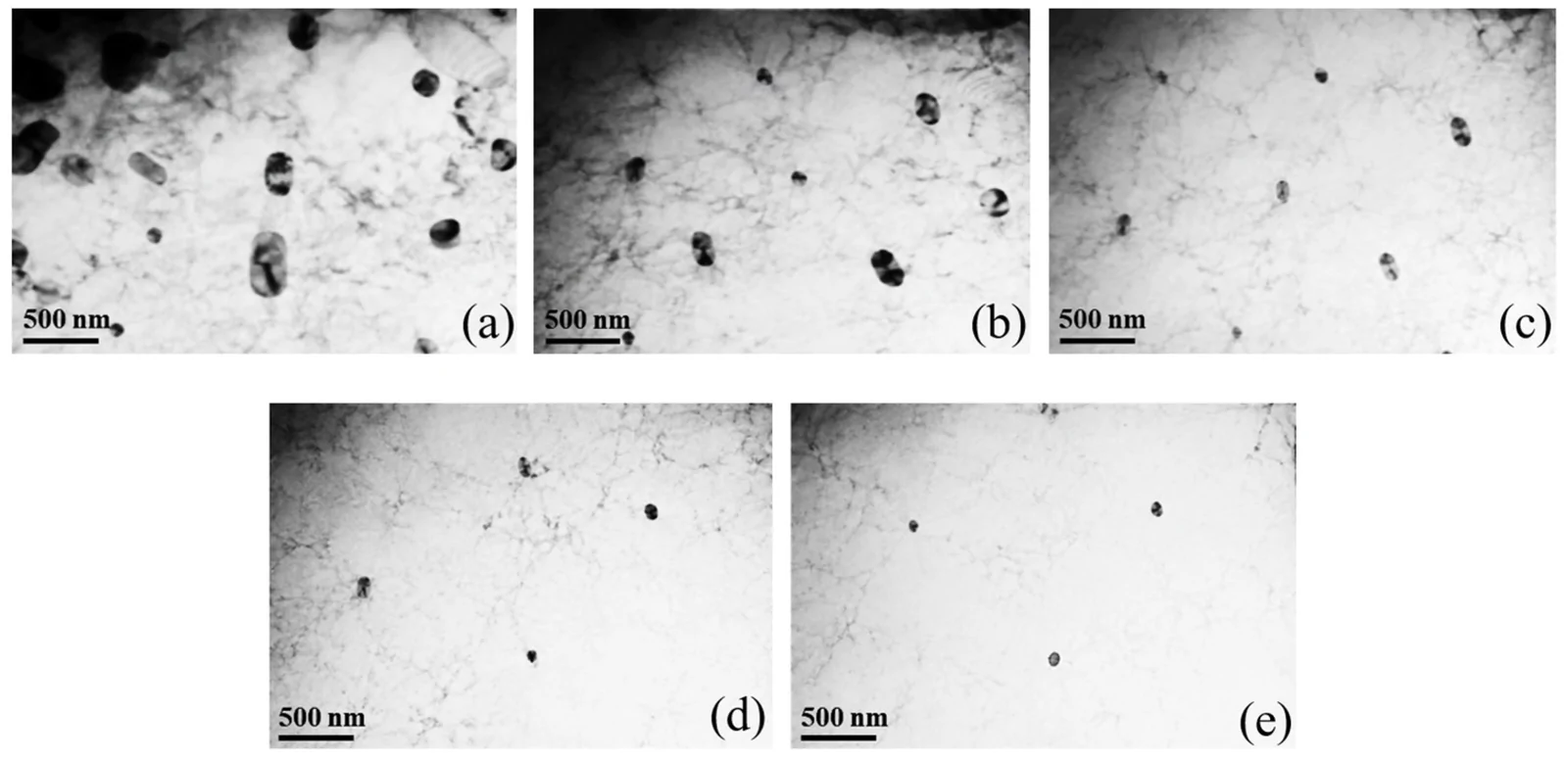
Bright-field TEM micrographs showing the evolution of residual carbides in the martensitic matrix of SCM440 steel: (**a**) AQ-1, (**b**) AQ-2, (**c**) AQ-3, (**d**) AQ-4, and (**e**) AH-80. Representative micrographs are shown for each condition.

Figure 5 shows bright-field TEM micrographs of the martensitic substructure and residual carbides after multiple quenching. Residual carbide particles were readily observed in AQ-1, with relatively coarse particles distributed within the martensitic matrix. With increasing cycle number, the carbides became progressively finer and less frequently observed, as evident from AQ-2 to AQ-4. In AH-80, only a small number of fine residual particles were observed, indicating the most extensive carbide dissolution among the investigated conditions. Importantly, the TEM observations confirm that nanoscale residual carbides persisted in AQ-3, AQ-4, and AH-80 despite being difficult to distinguish in the corresponding SEM micrographs (Figure 3). Thus, repeated austenitizing progressively reduced the size and population of residual carbides rather than resulting in their abrupt and complete disappearance. The quantitative evolution of carbide size, area fraction, and number density is presented in Table 3.

**Figure 6:**
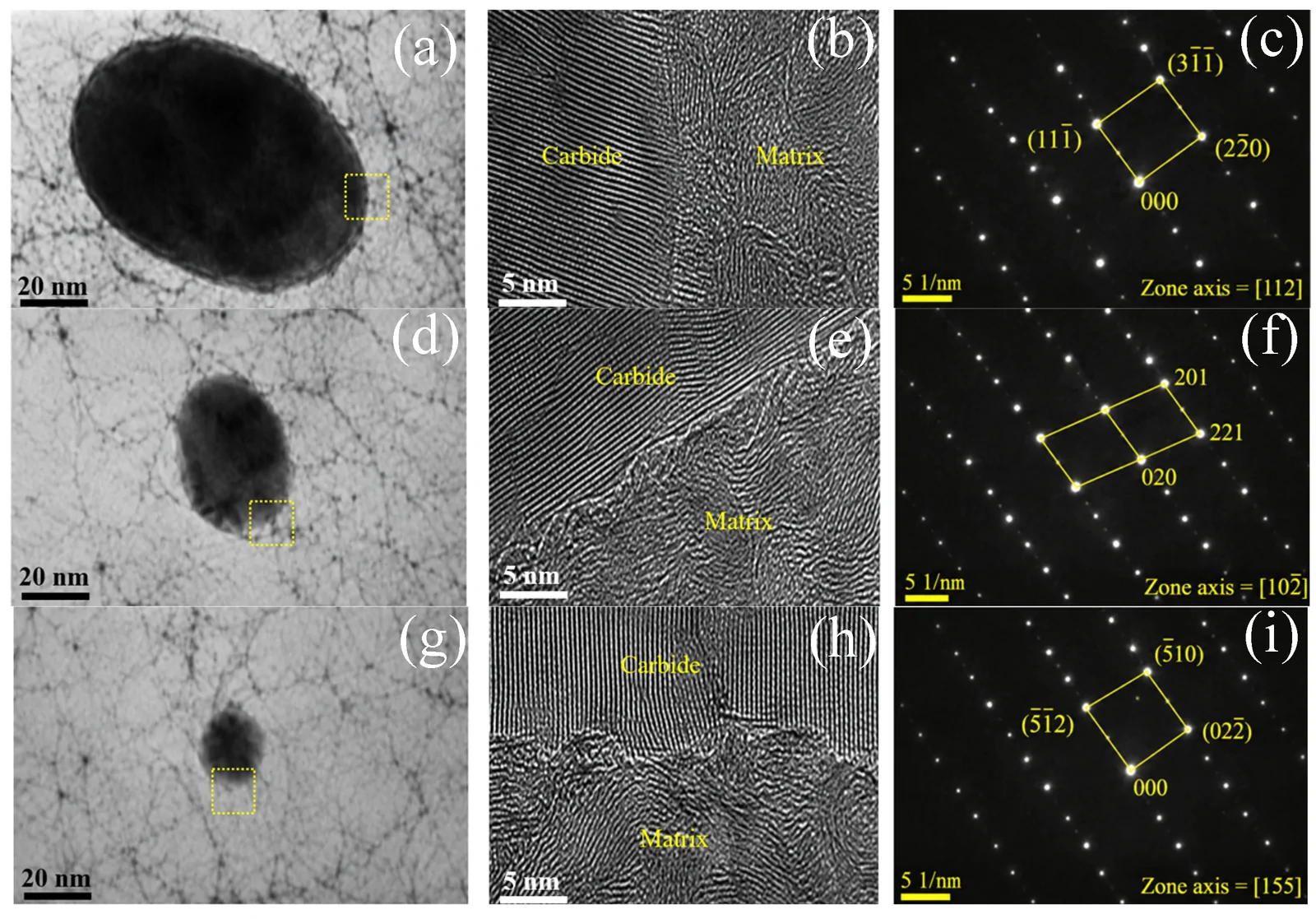
High-magnification TEM characterization of representative residual Fe_3_C particles in SCM440 steel for (**a**–**c**) AQ-1, (**d**–**f**) AQ-3, and (**g**–**i**) AH-80: (**a**,**d**,**g**) TEM images of individual residual carbides, (**b**,**e**,**h**) HRTEM images of the carbide/matrix interfacial regions indicated by the dashed boxes, and (**c**,**f**,**i**) corresponding electron diffraction patterns.

Figure 6 presents high-magnification TEM and HRTEM observations of representative residual carbides in AQ-1, AQ-3, and AH-80. The residual carbide particles were clearly distinguished from the surrounding martensitic matrix, and HRTEM images obtained from the selected regions revealed well-defined lattice fringes extending to the carbide/matrix interfaces. The corresponding SAED patterns exhibited distinct diffraction spots, confirming the crystalline nature of the residual carbides. These observations demonstrate that crystalline carbide particles remained in the martensitic matrix even after multiple quenching and prolonged austenitizing in AH-80. Together with the BF-TEM observations in Figure 5, the high-magnification TEM results indicate progressive changes in the residual carbide population with increasing thermal exposure.

**Figure 7:**
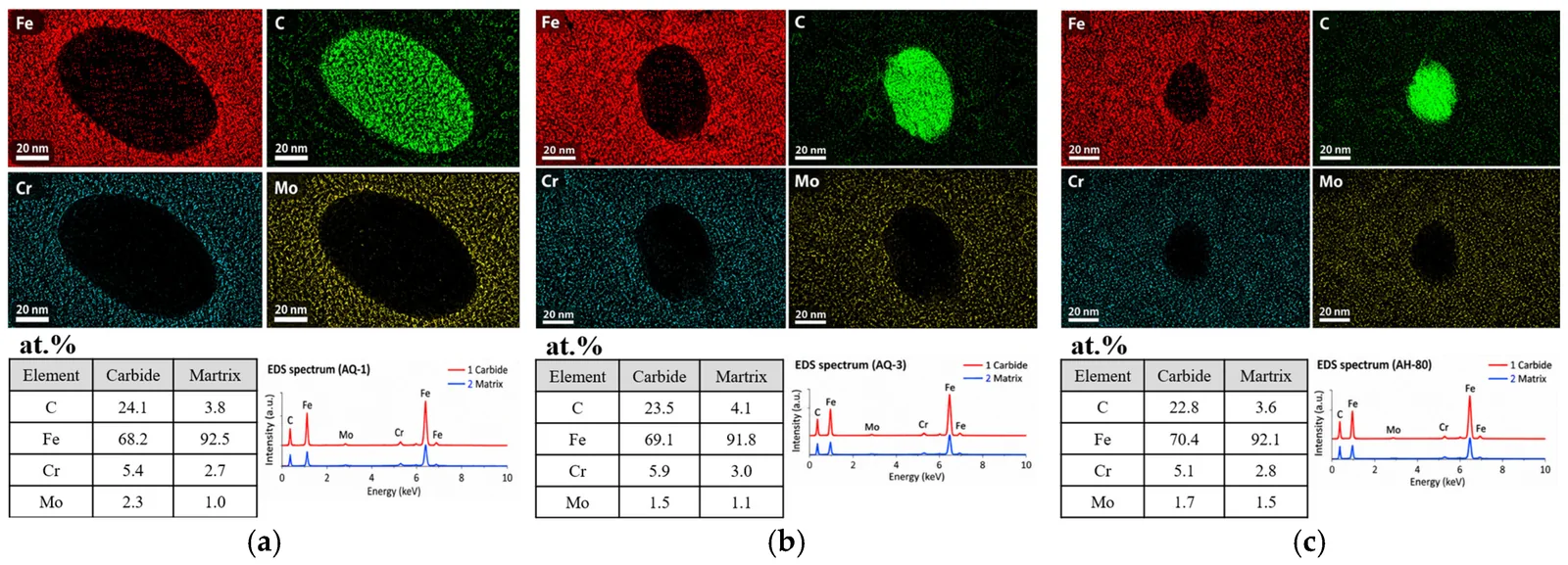
STEM-EDS characterization of residual carbide particles in SCM440 steel under (**a**) AQ-1, (**b**) AQ-3, and (**c**) AH-80 conditions. Elemental maps of Fe, C, Cr, and Mo, together with EDS spectra and compositional analyses of the carbide particles and surrounding martensitic matrix.

To further investigate the chemical characteristics of the residual carbides, STEM-EDS analyses were conducted for the AQ-1, AQ-3, and AH-80 conditions, as shown in Figure 7. The elemental distributions revealed carbon enrichment within the residual particles, while Fe remained the predominant metallic constituent, accompanied by minor amounts of Cr and Mo. These compositional characteristics, together with the crystallographic evidence obtained from the SAED patterns in Figure 6, are consistent with alloyed cementite (M_3_C-type carbide).

The presence of these carbide particles after quenching is consistent with incomplete carbide dissolution during austenitization. Under the rapid water-quenching conditions employed in this study, diffusion-controlled precipitation and growth of relatively coarse alloy carbides during cooling are expected to be strongly suppressed. Nevertheless, the possible formation of fine carbides through auto-tempering cannot be completely excluded, and the precise formation history of individual particles cannot be established from post-quenching characterization alone. The observed reductions in carbide size and area fraction with increasing thermal exposure are therefore consistent with progressive carbide dissolution during repeated austenitization, which may contribute to a reduction in grain-boundary pinning and the subsequent refinement-to-coarsening transition.

To quantitatively evaluate this evolution, the carbide size, area fraction, and number density were determined from the TEM micrographs, as summarized in Table 3.

All three quantitative parameters exhibited a systematic decrease with increasing thermal exposure. The mean carbide size decreased from 258.4 nm in AQ-1 to 120.7 nm in AQ-3 and further to 107.3 nm in AQ-4, while AH-80 exhibited the smallest mean carbide size of 76.3 nm. More importantly, the carbide area fraction decreased markedly from 7.39% in AQ-1 to 1.11% in AQ-3, 0.48% in AQ-4, and 0.23% in AH-80. The number density showed a corresponding decrease from 1.4821 to 0.5238 particles/μm^2^, confirming the progressive reduction in the residual carbide population. These quantitative results demonstrate a systematic reduction in the carbide population observed after quenching, which is consistent with progressive carbide dissolution during repeated or prolonged austenitization. Although the remaining particles became progressively finer, the pronounced reduction in carbide area fraction indicates that the overall particle population available for grain-boundary pinning was substantially diminished.

### 3.2. Competition Between Carbide Pinning and Grain-Growth Driving Force

The simultaneous reductions in carbide size and fraction have competing effects on Zener pinning because the pinning pressure depends on the ratio of particle fraction to particle radius. Therefore, the quantitative carbide characteristics in Table 3 were used to estimate the Zener pinning pressure ($P_{Z}$), which was compared with the estimated post-treatment capillary pressure ($P_{D}$) calculated from the final PAGS. This comparison was intended to assess the relative magnitudes of the two pressures rather than to independently predict grain-growth behavior. The resulting $P_{Z}$, $P_{D}$, and $P_{Z}$/$P_{D}$ values are summarized in Table 4.

The estimated $P_{Z}$ decreased from 0.429 MPa in AQ-1 to 0.045 MPa in AH-80, whereas $P_{D}$ reached its maximum of 0.235 MPa in AQ-3. The corresponding $P_{Z}$/$P_{D}$ ratio decreased from 2.616 to approximately 0.51. To assess the reliability of these estimates, uncertainties in carbide fraction, particle radius, and measured PAGS were propagated through the pressure equations (Table 4). The grain-boundary energy (*γ_GB_* = 0.5 J m^−2^) was treated as a fixed literature-based parameter. Both PZ and PD vary proportionally with *γ_GB_*, whereas their ratio ($P_{Z}$/$P_{D}$) remains independent of *γ_GB_* due to cancellation of the common term. The $P_{Z}$/$P_{D}$ ratio is therefore used as a simplified, post-treatment comparative metric for evaluating the relative balance between estimated carbide pinning and capillary driving pressures, rather than as a strict threshold for predicting grain refinement or coarsening. The decrease in $P_{Z}$ was primarily attributed to the reduction in carbide fraction from 7.39% in AQ-1 to 0.23% in AH-80, which outweighed the increase in pinning pressure expected from smaller particles. Thus, progressive carbide dissolution weakened Zener pinning. In contrast, $P_{D}$ peaked in AQ-3 owing to its minimum PAGS. However, because $P_{D}$ was calculated from the final grain size, both $P_{D}$ and $P_{Z}$/$P_{D}$ should be considered post-treatment comparative metrics rather than independent predictors of grain growth.

**Figure 8:**
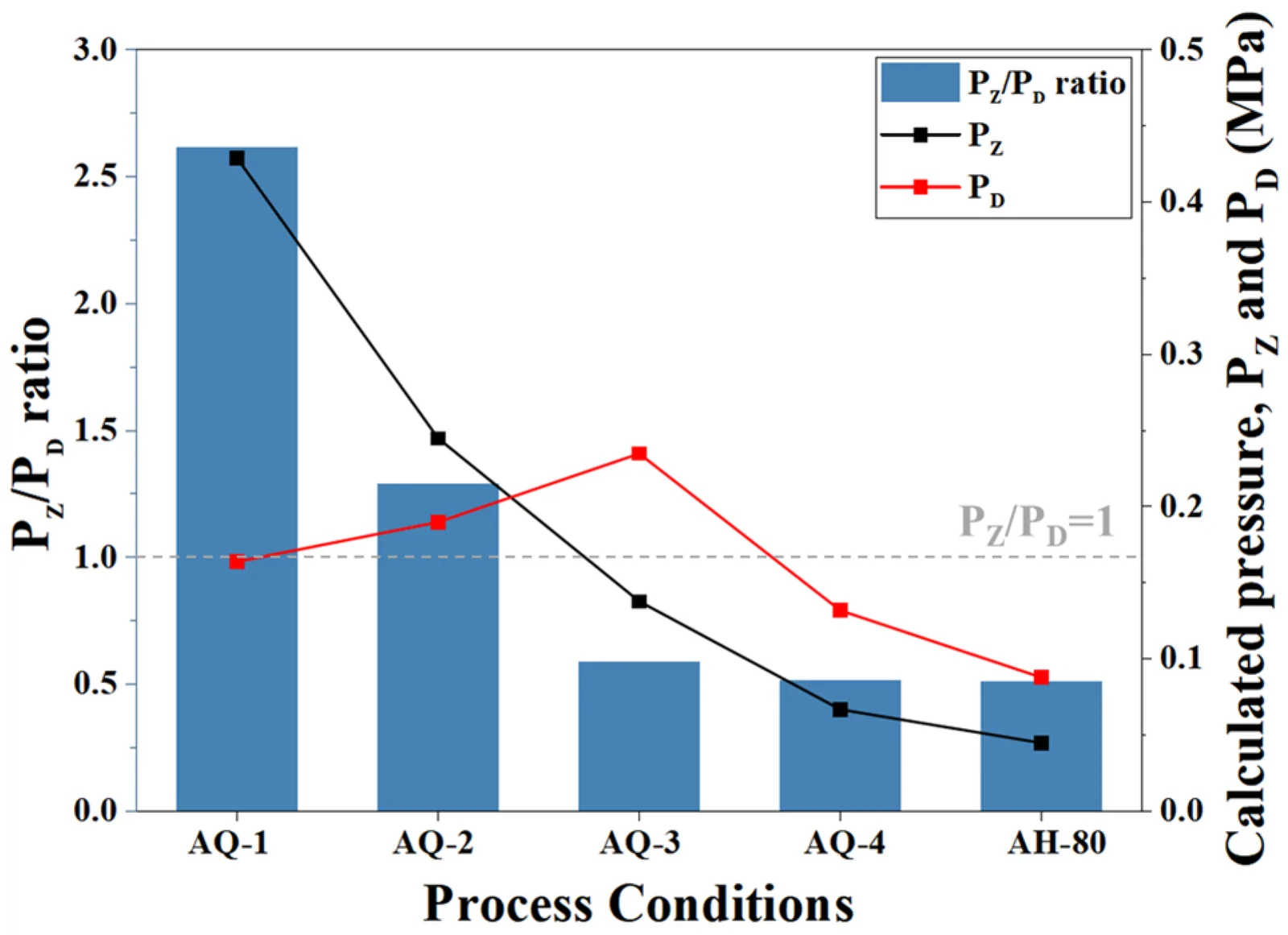
Evolution of the estimated Zener pinning pressure ($P_{Z}$), grain-growth driving pressure ($P_{D}$) and $P_{Z}/P_{D}$ ratio in SCM440 steel subjected to multiple quenching (AQ-1~AQ-4) and continuous austenitizing holding (AH-80).

Figure 8 compares the estimated pinning and driving pressures during repeated quenching. AQ-3 exhibited the minimum PAGS despite $P_{Z}$/$P_{D}$ falling below unity (0.587), indicating that the final-state ratio alone cannot predict grain coarsening. Continued refinement up to AQ-3 may reflect repeated austenite reformation at martensitic interfaces, a mechanism not directly captured by the pressure ratio. Previous studies have highlighted the roles of austenite nucleation during re-austenitization and carbide dissolution in controlling prior-austenite grain evolution [23,24,25]. These findings provide a mechanistic basis for interpreting the competing effects of repeated austenite reformation and progressively weakened carbide pinning observed in the present study.

However, this mechanism remains a proposed interpretation because austenite reformation was not directly examined by dilatometry or interrupted heat-treatment experiments. The subsequent coarsening in AQ-4 is consistent with a possible reduction in carbide pinning resistance, but its timing and underlying contributions cannot be established from the final-state pressure estimates. Therefore, the final PAGS evolution is interpreted as the combined outcome of repeated austenite reformation, carbide dissolution, and grain-boundary migration, rather than being governed by the calculated pressure ratio alone. Accordingly, the pressure comparison is regarded as a qualitative consistency analysis, and further validation through interrupted austenitization or time-resolved microstructural characterization is required to distinguish austenite reformation from subsequent grain growth.

Despite identical cumulative austenitizing times, AQ-4 exhibited finer PAGS than AH-80 (15.2 vs. 22.6 μm), suggesting a possible contribution from repeated austenite reformation. The refinement-to-coarsening transition may reflect competition between this process and progressively weakened carbide pinning, although direct mechanistic verification remains necessary.

**Figure 9:**
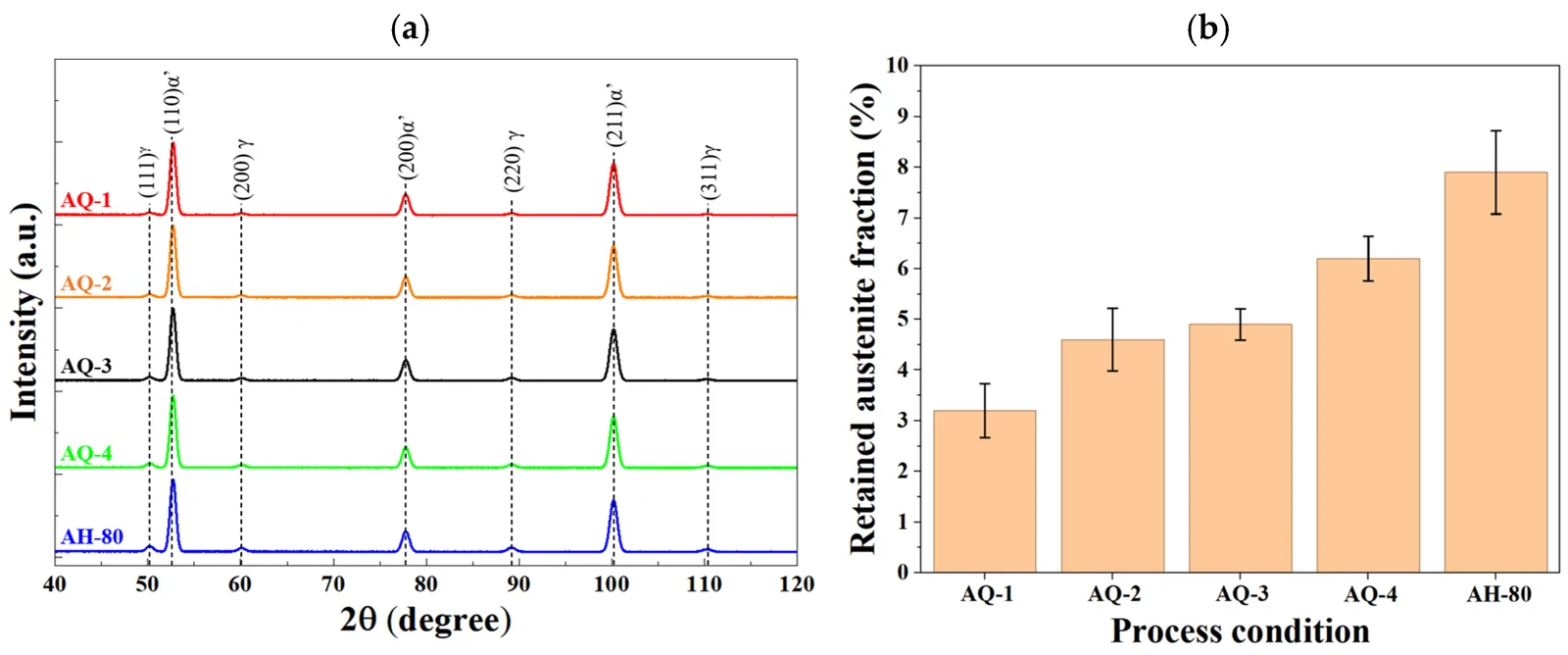
(**a**) X-ray diffraction patterns of SCM440 steel obtained using Co Kα radiation after repeated austenitizing and quenching (AQ-1–AQ-4) and continuous austenitizing holding (AH-80), and (**b**) corresponding retained-austenite fractions determined from XRD analysis. The indexed reflections correspond to martensite (*α*′) and retained austenite (*γ*). Error bars in (**b**) represent the standard deviations of five independent XRD measurements per condition (*n* = 5).

Figure 9 shows the XRD patterns and retained-austenite fractions of SCM440 steel under the different heat-treatment conditions. The diffraction peaks were indexed with reference to the standard diffraction patterns of α-Fe (JCPDS No. 00-006-0696) and *γ*-Fe (JCPDS No. 00-052-0512). As shown in Figure 9a, all specimens exhibited dominant martensitic *α*′ reflections together with relatively weak *γ*-austenite reflections. The major martensitic peaks were observed at approximately 52.0°, 77.6°, and 100.0° in 2θ, corresponding to the (110)*α*′, (200)*α*′, and (211)*α*′ reflections, respectively. The retained-austenite peaks were identified at approximately 50.5°, 59.9°, 89.0°, and 110.5°, corresponding to the (111)*γ*, (200)*γ*, (220)*γ*, and (311)*γ* reflections, respectively. These *γ* reflections confirm the presence of retained austenite after quenching.

As shown in Figure 9b, the retained-austenite fraction progressively increased from 3.2% in AQ-1 to 4.6% in AQ-2 and 4.9% in AQ-3, followed by further increases to 6.2% in AQ-4 and 7.9% in AH-80. This trend coincided with the reduction in residual carbide population independently observed by TEM, rather than providing direct XRD evidence of carbide dissolution. Carbide dissolution during repeated or prolonged austenitizing may increase the carbon and alloying elements dissolved in austenite, potentially enhancing its stability and retention after quenching. However, because solute redistribution was not directly quantified, the observed increase in retained austenite should be interpreted as consistent with, rather than direct evidence of, progressive carbide dissolution.

### 3.3. Mechanical Properties and Grain-Refinement Mechanism

**Figure 10:**
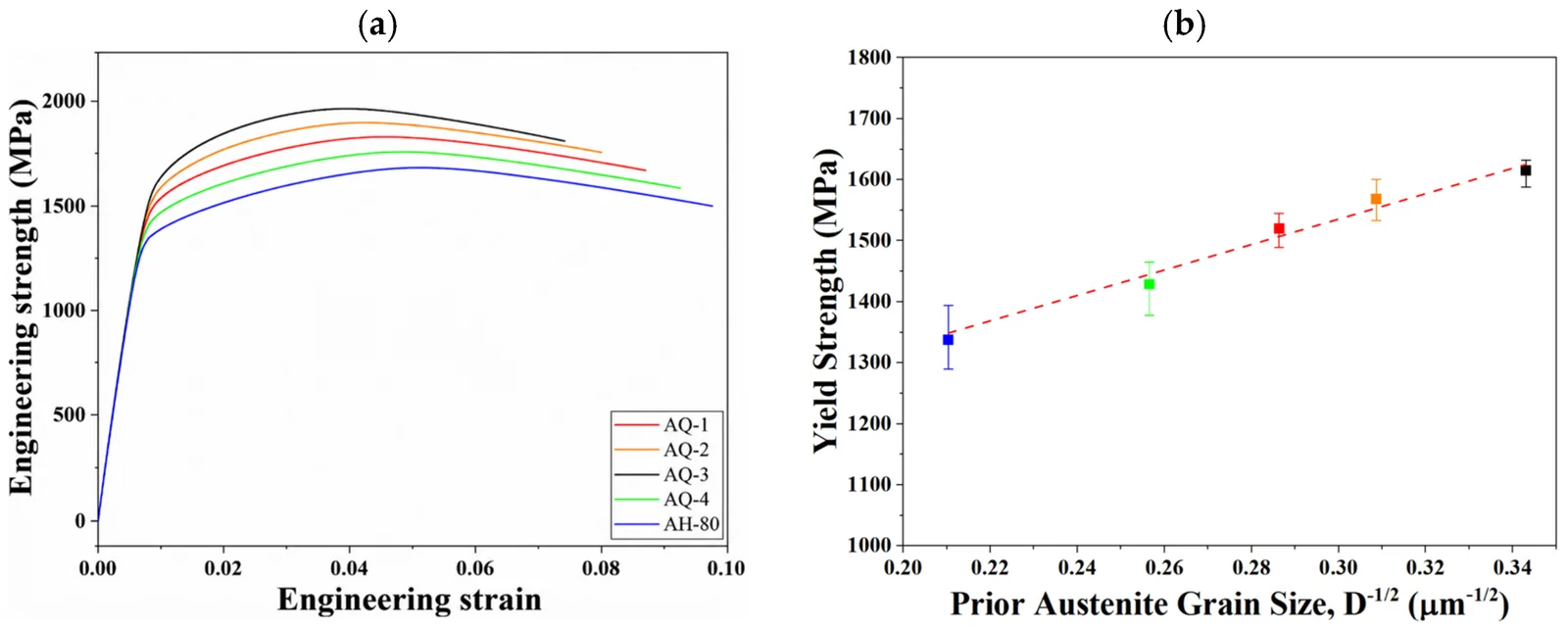
Mechanical response of SCM440 steel under different heat-treatment conditions: (**a**) average engineering stress–strain curves and (**b**) relationship between yield strength and the inverse square root of the prior-austenite grain size (PAGS^−1/2^).

Figure 10 presents the average engineering stress–strain curves and Hall–Petch-type relationship of SCM440 steel under different heat-treatment conditions, while the corresponding tensile properties, including yield strength, ultimate tensile strength, uniform elongation, and total elongation, are summarized in Table 5.

As shown in Figure 10a, the strength progressively increased from AQ-1 to AQ-3, with the average yield strength (YS) increasing from 1520 to 1615 MPa and the average ultimate tensile strength (UTS) from 1810 to 1932 MPa. AQ-3 therefore exhibited the highest strength, coinciding with the minimum PAGS of 8.5 μm. Further cycling resulted in a decrease in strength, with average YS and UTS decreasing to 1429 and 1755 MPa in AQ-4, respectively, while AH-80 exhibited the lowest values of 1338 and 1662 MPa. The tensile results revealed a strength–ductility trade-off, with uniform and total elongation decreasing from 4.3% and 8.7% in AQ-1 to 4.0% and 7.4% in AQ-3 as strength increased. Conversely, AQ-4 and AH-80 exhibited improved ductility at lower strengths, with AH-80 showing the highest elongations (5.0% and 9.8%), indicating that microstructural evolution influenced both strength and ductility.

As shown in Figure 10b, YS exhibited a strong linear correlation with PAGS^−1^/^2^, described by YS = 910.449 + 2084.344PAGS^−1^/^2^ (R^2^ = 0.979). Although the corresponding unweighted regression was statistically significant (*p* = 0.003), this relationship should be interpreted as a correlation rather than direct evidence of a Hall–Petch strengthening mechanism, considering the limited number of data points and concurrent changes in retained-austenite fraction and martensitic substructure. Although YS showed a strong correlation with PAGS^−1^/^2^, other microstructural changes accompanied grain refinement. From AQ-1 to AQ-3, the EBSD-derived effective grain size decreased from 1.28 to 0.78 μm, while KAM increased from 1.05° to 1.62°, alongside a 6.3% increase in YS. These concurrent changes, together with variations in retained-austenite fraction, suggest that strength evolution cannot be attributed exclusively to PAGS refinement.

**Figure 11:**
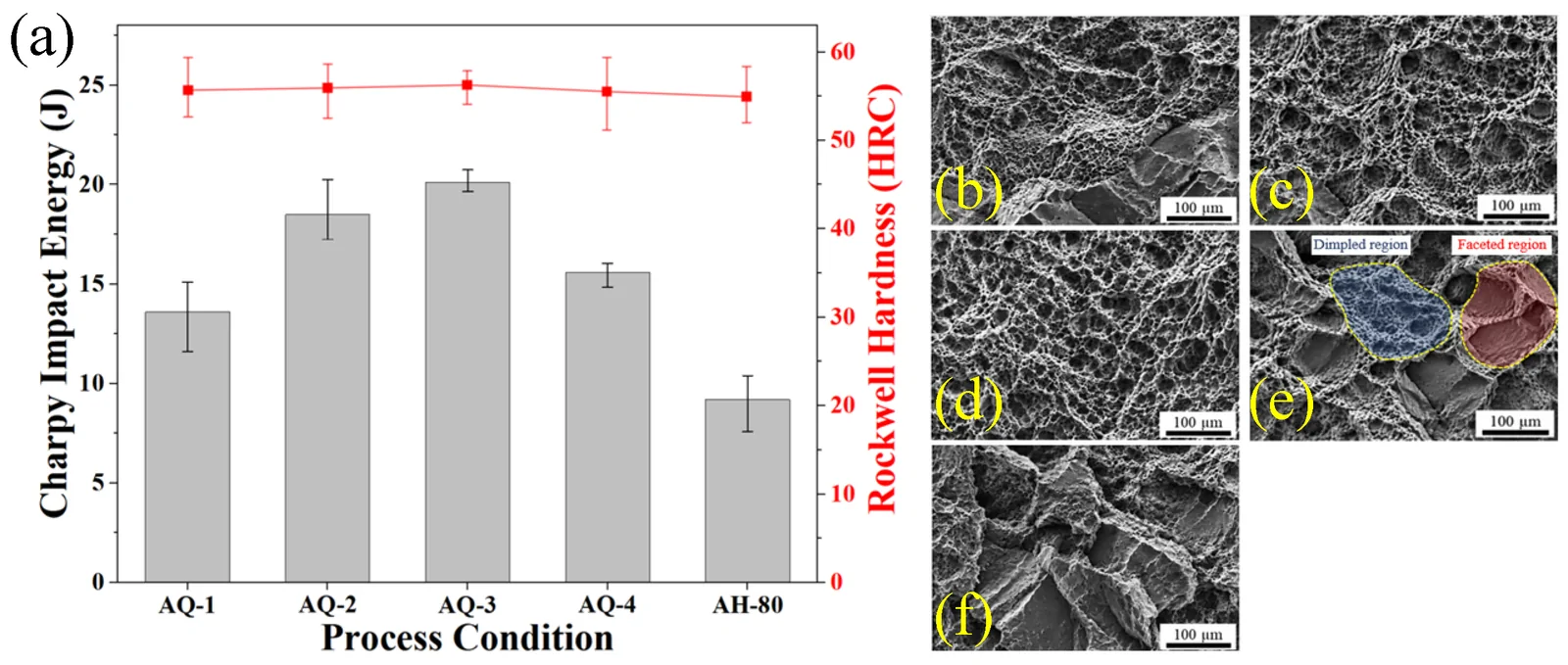
Charpy impact properties and fracture behavior of SCM440 steel under different heat-treatment conditions: (**a**) Charpy impact energy and Rockwell hardness, and representative fracture-surface SEM images of (**b**) AQ-1, (**c**) AQ-2, (**d**) AQ-3, (**e**) AQ-4, and (**f**) AH-80. Error bars for Charpy impact energy represent the standard deviation of seven independent measurements (*n* = 7).

Figure 11 presents the impact properties and representative fracture morphologies of SCM440 steel under different heat-treatment conditions. As shown in Figure 11a, the mean Charpy impact energy increased from 13.60 ± 1.44 J in AQ-1 to 18.50 ± 0.97 J in AQ-2, reaching a maximum of 20.10 ± 0.37 J in AQ-3, before decreasing to 15.60 ± 0.40 J in AQ-4 and 9.20 ± 1.19 J in AH-80 (mean ± SD, *n* = 7). Games–Howell post hoc comparisons indicated significant differences between AQ-3 and AQ-2 (*p* = 0.023), AQ-3 and AQ-4 (*p* < 0.001), and AQ-2 and AQ-4 (*p* < 0.001). In contrast, Rockwell hardness remained relatively unchanged, possibly due to limited differences in dissolved carbon content despite carbide dissolution observed by TEM. Meanwhile, variations in PAGS and martensitic substructure may have contributed to the appreciable changes in tensile strength and impact resistance.

The fracture morphologies in Figure 11b–f illustrate the variation in impact energy across the heat-treatment conditions. AQ-1 [Figure 11b] exhibited mixed dimpled and faceted regions, while AQ-2 [Figure 11c] showed more extensive dimpling. AQ-3 [Figure 11d] displayed predominantly fine dimples and a tortuous fracture surface, whereas AQ-4 [Figure 11e] exhibited more pronounced flat facets. AH-80 [Figure 11f] showed larger faceted regions with limited dimpling, consistent with its lowest impact energy. Semi-quantitative analysis yielded faceted area fractions of 10.62%, 3.73%, 1.49%, 10.84%, and 22.55% for AQ-1, AQ-2, AQ-3, AQ-4, and AH-80, respectively. The lowest faceted fraction in AQ-3 coincided with the highest impact energy and finest PAGS, suggesting that microstructural refinement and reduced faceted fracture characteristics may be associated with enhanced impact resistance. Conversely, the subsequent reduction in impact energy in AQ-4 and AH-80 may be associated with prior-austenite grain coarsening and the increased proportion of faceted fracture regions, which are consistent with reduced resistance to crack propagation. The superior mechanical properties of AQ-3 may be associated with its refined PAGS (8.5 μm), martensitic substructure, and reduced faceted fracture fraction (1.49%). In contrast, grain coarsening and increased faceted fracture in AQ-4 and AH-80 coincided with reduced strength and impact energy, despite higher retained-austenite fractions.

## 4. Conclusions

The effects of multiple quenching on the microstructure and mechanical properties of SCM440 steel were investigated. The main conclusions are as follows:

1. The PAGS decreased from 12.2 μm in AQ-1 to a minimum of 8.5 μm in AQ-3 and subsequently increased to 15.2 μm in AQ-4. AH-80 exhibited the largest PAGS of 22.6 μm despite the same cumulative austenitizing time as AQ-4, demonstrating a clear refinement-to-coarsening transition during multiple quenching.
2. Meanwhile, the projected carbide area fraction decreased from 7.39% in AQ-1 to 0.23% in AH-80, suggesting progressive carbide dissolution with increasing thermal exposure, although the precise formation history of individual particles could not be established.
3. The estimated Zener pinning pressure decreased from 0.429 to 0.045 MPa, while $P_{Z}$/$P_{D}$ declined from 2.616 to approximately 0.51. Grain refinement continued up to AQ-3, suggesting a possible contribution from repeated austenite reformation. However, these pressure estimates relied on simplified models, and re-austenitization was not directly observed. Progressive carbide dissolution may have weakened grain-growth resistance, contributing to subsequent coarsening.
4. Among the investigated as-quenched conditions, AQ-3 provided the finest microstructure and highest measured tensile strength and Charpy impact energy, whereas these properties decreased in AQ-4 and AH-80. These findings suggest limiting repeated-quenching cycles when progressive carbide dissolution reduces resistance to grain growth.

## Figures and Tables

**Table 1:** Chemical composition of SCM 440 steel (wt.%).

wt.%	C	Si	Mn	Cr	Ni	Mo	Cu	P	S	Fe
SCM440	0.412	0.235	0.747	1.023	0.244	0.241	0.104	0.008	0.003	Balance.

**Table 2:** Quantitative EBSD characteristics of SCM440 steel under different heat-treatment conditions.

Condition	Effective Grain Size (μm)	HAGB (%)	LAGB (%)	Avg. KAM (°)
AQ-1	1.28 ± 0.14	35.2 ± 3.1	64.8 ± 3.1	1.05 ± 0.12
AQ-2	1.02 ± 0.11	39.6 ± 3.4	60.4 ± 3.4	1.28 ± 0.14
AQ-3	0.78 ± 0.09	45.8 ± 3.7	54.2 ± 3.7	1.62 ± 0.16
AQ-4	0.85 ± 0.10	42.1 ± 3.5	57.9 ± 3.5	1.44 ± 0.15
AH-80	1.55 ± 0.17	32.4 ± 2.9	67.6 ± 2.9	0.88 ± 0.10

**Table 3:** Quantitative characteristics of residual carbides in SCM440 steel subjected to multiple quenching. Values are presented as mean ± standard deviation based on five TEM observation fields per condition (*n* = 5).

Condition	Carbide Size (nm)	Area Fraction (%)	Number Density (Particles/μm^2^)
AQ-1	258.4 ± 50.5	7.39 ± 0.82	1.4821 ± 0.0105
AQ-2	171.1 ± 38.7	2.79 ± 0.36	1.2594 ± 0.0212
AQ-3	120.7 ± 25.4	1.11 ± 0.17	0.8925 ± 0.0094
AQ-4	107.3 ± 10.1	0.48 ± 0.09	0.6123 ± 0.0059
AH-80	76.3 ± 7.8	0.23 ± 0.05	0.5238 ± 0.1245

**Table 4:** Estimated post-treatment capillary pressure ($P_{D}$), Zener pinning pressure ($P_{Z}$), and their ratio ($P_{Z}$/$P_{D}$) for SCM440 steel under different heat-treatment conditions.

Condition	Grain-Growth Driving Pressure $P_{D}$ (MPa)	Zener Pinning $P_{Z}$ (MPa)	*P_Z_*/*P_D_*
AQ-1	0.164 ± 0.021	0.429 ± 0.064	2.616 ± 0.515
AQ-2	0.190 ± 0.022	0.245 ± 0.039	1.289 ± 0.254
AQ-3	0.235 ± 0.028	0.138 ± 0.023	0.587 ± 0.120
AQ-4	0.132 ± 0.018	0.067 ± 0.012	0.515 ± 0.116
AH-80	0.088 ± 0.011	0.045 ± 0.009	0.511 ± 0.112

**Table 5:** Tensile properties of SCM440 steel under different heat-treatment conditions. Values are expressed as mean ± standard deviation (SD).

Condition	Yield Strength (MPa)	Ultimate Tensile Strength (MPa)	Uniform Elongation (%)	Total Elongation (%)
AQ-1	1520 ± 18.2	1810 ± 21.7	4.3 ± 0.4	8.7 ± 0.4
AQ-2	1569 ± 22.1	1872 ± 26.4	4.2 ± 0.3	8.0 ± 0.5
AQ-3	1615 ± 14.3	1932 ± 17.1	4.0 ± 0.2	7.4 ± 0.3
AQ-4	1429 ± 28.2	1755 ± 34.6	4.8 ± 0.3	9.3 ± 0.4
AH-80	1338 ± 33.8	1662 ± 42.0	5.0 ± 0.1	9.8 ± 0.5

## Data Availability

The original contributions presented in this study are included in the article/Supplementary Material. Further inquiries can be directed to the corresponding author.

## Supplementary Materials

The following supporting information can be downloaded at https://www.mdpi.com/article/10.3390/ma19214572/s1, Figure S1: Distributions of carbide equivalent-circle diameters in SCM440 steel subjected to different heat-treatment conditions: (a) AQ-1, (b) AQ-2, (c) AQ-3, (d) AQ-4, and (e) AH-80. The histograms illustrate the progressive reduction in carbide size with increasing quenching cycles and continuous austenitizing. A uniform bin width of 10 nm was applied to all conditions. Figure S2: Continuous cooling transformation (CCT) diagram of SCM440 steel calculated using JmatPro v.14 at an austenitizing temperature of 850 °C. The diagram illustrates the predicted ferrite, pearlite, and bainite transformation boundaries (0.1% and 99.9%) together with continuous cooling curves at cooling rates of 0.1, 1, 10, and 100 °C/s.

## Abbreviations

The following abbreviations are used in this manuscript:

AQ	Austenitization and quenching
AH	Austenitization and holding
PAG	Prior-austenite grain
PAGS	Prior-austenite grain size
EBSD	Electron backscatter diffraction
SEM	Scanning electron microscopy
XRD	X-ray diffraction
UTS	Ultimate tensile strength
YS	Yield strength
