# Supplementary material for: Refinement-to-Coarsening Transition During Multiple Quenching of SCM440 Steel: Competing Roles of Austenite Reformation and Carbide Dissolution
Source: Materials (Basel). 2026 Oct 29;19(21):4572. doi: 10.3390/ma19214572 (PMC13764764; doi:10.3390/ma19214572)
Supplement: Supplementary file 1 [file materials-19-04572-s001.zip › materials-4614410-supplementary.pdf]

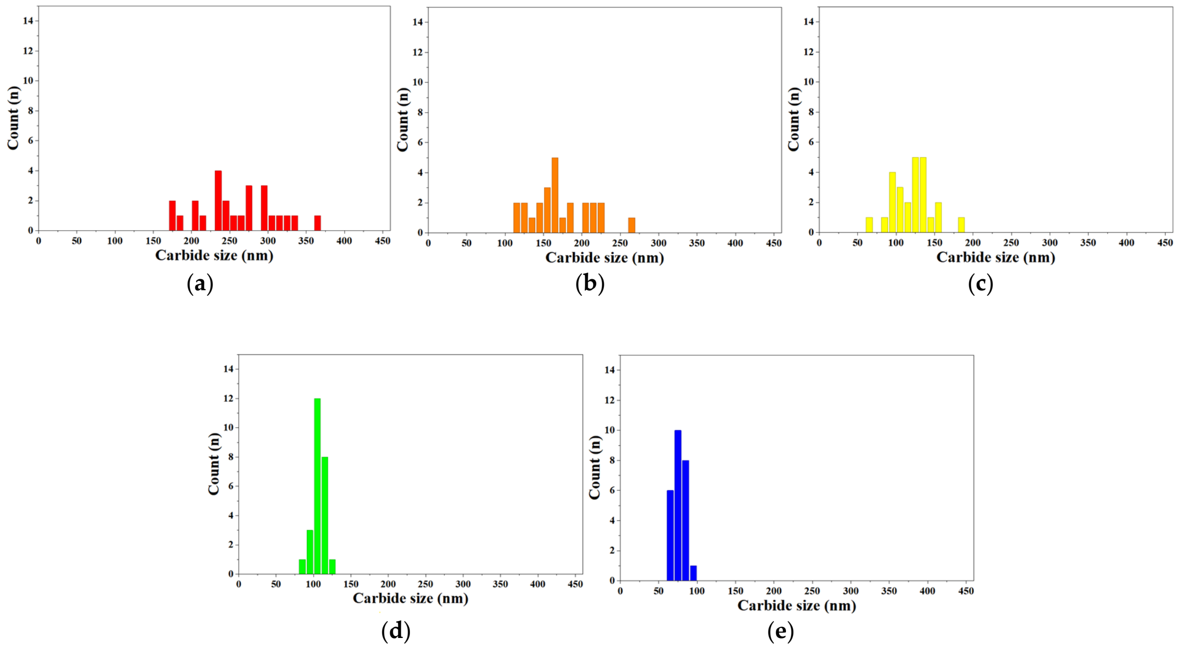

Supplementary Figure S1. Distributions of carbide equivalent-circle diameters in SCM440 steel subjected to different heat-treatment conditions: (a) AQ-1, (b) AQ-2, (c) AQ-3, (d) AQ-4, and (e) AH-80. The histograms illustrate the progressive reduction in carbide size with increasing quenching cycles and continuous austenitizing. A uniform bin width of 10 nm was applied to all conditions.

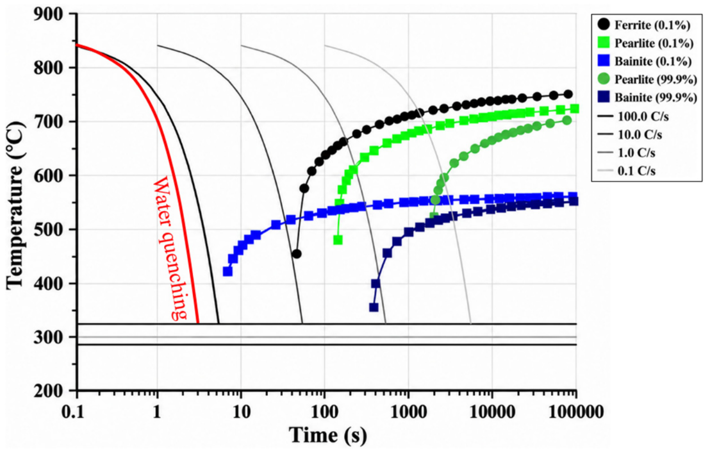

Supplementary Figure S2. Continuous cooling transformation (CCT) diagram of SCM440 steel calculated using JmatPro v.14 at an austenitizing temperature of 850 °C. The diagram illustrates the predicted ferrite, pearlite, and bainite transformation boundaries (0.1% and 99.9%) together with continuous cooling curves at cooling rates of 0.1, 1, 10, and 100 °C/s.
